# Nonsurgical Intervention in a Preeclamptic Patient with Spontaneous Spinal Epidural Hematoma

**DOI:** 10.1155/2018/5879481

**Published:** 2018-11-07

**Authors:** Michelle Nguyen, Maria Raquel Kronen, Alex Nhan, Antonio Liu

**Affiliations:** ^1^Department of Obstetrics and Gynecology, White Memorial Medical Center, Los Angeles, CA 90033, USA; ^2^University of Central Florida College of Medicine, Orlando, FL 32827, USA; ^3^Department of Neurology, White Memorial Medical Center, Los Angeles, CA 90033, USA

## Abstract

**Background:**

Spontaneous epidural hematoma (SEH) is a rare finding in pregnancy, especially since most pregnant women do not have risk factors for developing SEH. The presence of epidural anesthesia can delay the diagnosis of SEH in pregnant patients. Immediate surgical decompression is the current standard of care for treating SEH.

**Case Presentation:**

We present the case of a 37-year-old pregnant woman with preeclampsia with severe features who developed neurological deficits that were initially attributed to her epidural anesthesia. She was eventually found to have SEH with spinal stenosis at T5-T6 on MRI. Oral antihypertensives were used to keep the patient's blood pressures within normal limits, and she subsequently had complete resolution of her neurological symptoms and her SEH on imaging.

**Conclusion:**

Preeclampsia may contribute to the development of SEH in pregnancy, and strict blood pressure control may potentially provide a safe and effective alternative to neurosurgery for these patients.

## 1. Introduction

Spontaneous epidural hematoma (SEH) is a particularly rare and devastating complication in the peripartum period. SEH in pregnant patients may remain undetected for a significant amount of time as these patients are less likely to have predisposing factors for SEH, such as anticoagulation, arteriovenous (AV) malformations, hemophilia, or trauma [1]. Furthermore, the administration of epidural anesthesia can confound the clinical picture, posing another challenge in making the diagnosis. Prompt recognition of SEH in laboring patients is extremely important because urgent treatment is required to prevent long-term neurological damage and other associated complications, including seizure, placental abruption, hepatic rupture, and coagulopathy [2]. The standard treatment for SEH is immediate surgical decompression, as demonstrated in the rare cases of SEH in pregnancy that have been reported in the literature [3–7].

While SEH may present under a variety of conditions in the intrapartum period [8–14], SEH that is diagnosed postpartum in preeclamptic patients is especially challenging to treat. Maintaining the perfusion pressure of the spinal cord is important because decreased blood pressure can lead to decreased spinal cord blood flow, which could further compromise the segment of spinal cord that has already been injured by the spontaneous hemorrhage [15, 16]. However, lowering blood pressure is important in order to reduce the preeclampsia-associated risks of seizures, stroke, hepatic rupture, and renal injury. The ACOG Task Force on Hypertension in Pregnancy recommends antihypertensive therapy in the postpartum period when blood pressure is persistently higher than 150 mm Hg systolic or 100 mm Hg diastolic [17]. This case study describes a preeclamptic patient with SEH who was conservatively managed with antihypertensive medication in the postpartum period to maintain blood pressures within a normal range (i.e., systolic <140, diastolic <90, below current literature recommendations) and made a full neurological recovery without invasive surgical intervention.

## 2. Case Report

A 37-year-old woman with a history of chronic back pain and sciatica presented to our teaching hospital at 36.5 weeks' gestation in early labor. At the time of presentation, she was noted to have acute onset of mild-range elevated blood pressures (140s-150s/90s) with a urine protein-to-creatinine ratio of 0.37, consistent with a diagnosis of preeclampsia. Six hours after admission, her blood pressures progressed to severe-range, with a maximum of 195/105. Per protocol, she was given IV labetalol and MgSO_4_ for preeclampsia with severe features. Shortly thereafter, the patient retrospectively reported that she began to have mid-back pain along with numbness, tingling, and weakness in her right lower extremity, but she did not report these symptoms initially to her healthcare team, as she was more concerned about her pelvic pain with contractions. Approximately 3 hours after the onset of her neurological symptoms, a labor epidural was administered to help control her contraction pain and blood pressures. The epidural catheter was placed uneventfully at L3-L4 with the tip threaded to the maximum height of T11. As the epidural was being placed, the patient then reported to the anesthesiologist that she had been feeling weak. The patient was noted to appear lethargic on exam, but she was able to sit up with minimal assistance for her labor epidural. Therefore, her weakness was attributed to labor. She progressed to complete cervical dilation and had a vaginal delivery with vacuum assistance due to a 5-minute prolonged deceleration on FHT.

The patient continued to complain of leg weakness after delivery. At 14 hours postpartum, the nurse encouraged the patient to attempt ambulation. However, even with her best efforts, the patient was unable to move her body from a distinct line below her breasts down to her toes. She also noticed numbness, burning, and electrical sensations to light touch from that line down to her toes. At this time the resident team was notified, and a Foley catheter was inserted. There was low suspicion for magnesium toxicity as she had intact reflexes with no complaints of shortness of breath, and her magnesium level was 5.9. She still had mild-range elevated blood pressures at the time, and she remained on IV magnesium for 24 hours postpartum.

**Figure 1:**
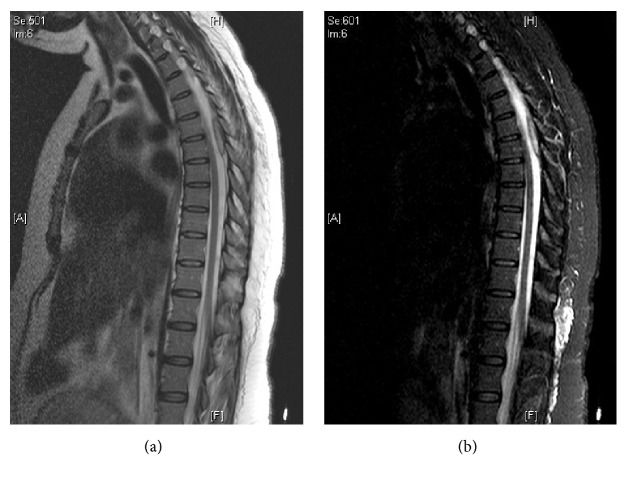
(a, b) MRI images at 14 hours postpartum showing a spinal epidural hematoma measuring 3.5 cm x 0.4 cm. There is also spinal stenosis at T5-T6 due to extrinsic mass effect of the epidural hemorrhage.

A stat CT scan of the head without contrast resulted in normal findings with no evidence of stroke. MRI of the spine showed a fluid sac suggestive of epidural blood, measuring 3.5 cm in the craniocaudal plane and 0.4 cm in the anteroposterior plane. There was also a mild-to-moderate degree of spinal stenosis at T5-T6 due to extrinsic mass effect of the epidural hemorrhage but no direct spinal cord compression (Figures 1(a) and 1(b)). The patient was immediately started on IV dexamethasone 4 mg q6h. Upon evaluation by neurosurgery, the patient was not considered to be a surgical candidate because the MRI showed no clear evidence of spinal cord hemorrhage or spinal cord compression.

**Figure 2:**
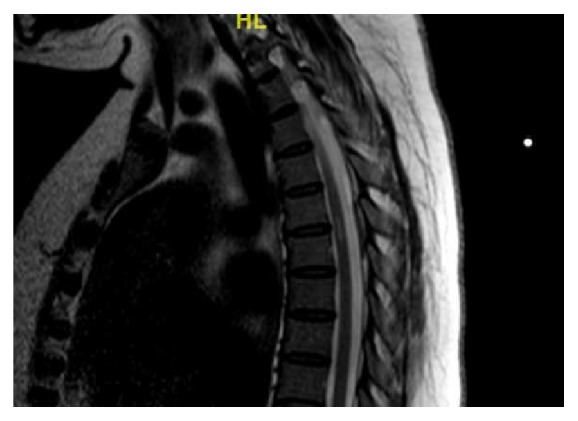
MRI T2 sagittal view of epidural blood collection which has decreased in size to 2-3 mm.

On the morning of postpartum day #1, the patient remained with paresthesia in her lower extremities and flaccid paralysis from the waist down, but she was able to wiggle her toes. Her blood pressures were predominately normal (120-140/80-90) with a few mild-range elevated blood pressures. Per protocol, she was kept on IV magnesium for seizure prophylaxis until she was 24 hours postpartum. Diffusion-weighted imaging of the spine later that day showed an epidural lesion with a hemosiderin ring that had decreased in size to 2-3 mm in maximal depth, suggestive of a resolving epidural hematoma when compared to the most recent MRI (Figure 2).

On postpartum day #2, the patient was started on PO nifedipine XL 30 mg daily to consistently maintain her blood pressures within normal range. Her mobility improved with demonstrated flexion and extension at the hips bilaterally, in addition to return of normal sensation in her lower extremities.

The patient's movements and sensation continued to improve day by day while she was kept on IV dexamethasone and PO nifedipine. By postpartum day #4, the patient was ambulating with a walker and had good bladder and bowel control. On postpartum day #6, the patient was ambulating without assistance and reported complete resolution of her pain in the back and lower extremities. She was discharged home in stable condition.

**Figure 3:**
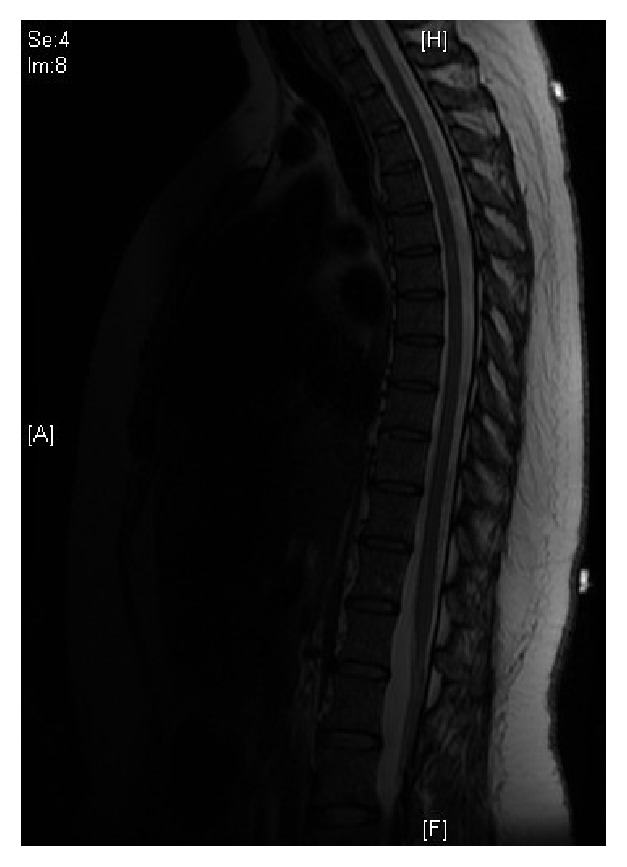
MRI at 6 weeks postpartum showing complete resolution of the spontaneous epidural hematoma.

A follow-up MRI 6 weeks later showed complete resolution of the spinal epidural hematoma (Figure 3). At the time, she was still ambulating independently and had full control of her bladder and bowel function.

## 3. Discussion

The underlying pathogenesis of preeclampsia involves abnormalities in the placental vasculature, which lead to decreased circulating levels of VEGF (vascular endothelial growth factor) and other angiogenic growth factors. Decreased VEGF in turn causes endothelial cell dysfunction, which leads to vasoconstriction (i.e., spasms in the vascular smooth muscle) and elevated blood pressures [18]. Preeclampsia may have played a role in the development of SEH. Our patient first noticed back pain along with numbness, tingling, and weakness in her right lower extremity around the same time that her blood pressures progressed from mild-range (i.e., systolic 140-159 mm Hg, diastolic 90-109 mm Hg) to severe-range (i.e., systolic ≥160, diastolic ≥110). We suspect that the patient's increased blood pressures may have initiated her SEH at the T5-T6 region, which subsequently led to a transient extrinsic compression of the spinal cord and eventually cord edema. Epidural placement was likely a coincident rather than causative factor, given that the patient's neurological symptoms began prior to epidural anesthesia, and the lesion occurred above the level of epidural placement. Although epidural anesthesia made the diagnosis of SEH more difficult, the patient's cord edema was fortunately reversible with IV dexamethasone. Consistently maintaining her blood pressures within normal range (120-140/80-90) using PO antihypertensive medication likely also facilitated neurologic recovery.

Several challenges emerged in the diagnosis and treatment of this patient. First of all, our patient was a relatively healthy pregnant woman in her 30s with no preexisting medical conditions, prior surgeries, or trauma history. In contrast, SEH typically presents in the fourth or fifth decade of life, and it is more likely to occur in males [19]. The epidemiology of SEH is in part secondary to natural history of AV malformations, which are among the most significant predisposing factors. Spinal AV malformations, particularly dural AV fistulas, affect males more often than females, with the average age at diagnosis being 50s-60s [20]. This makes the presence of AV malformations in obstetric patients significantly less likely, and the diagnosis of ruptured SEH easy to miss. This is clinically significant because AV malformations have the potential to precipitate spontaneous hemorrhage and/or cord ischemia, and pregnancy increases the risk of hemorrhage from AV malformations [21].

## 4. Conclusion

Our patient achieved excellent outcomes from strict blood pressure control without undergoing invasive neurosurgery. The standard of care of neurosurgical treatment of SEH may warrant revisiting in obstetric patients whose cases are complicated by preeclampsia, in light of this study.
